# Chemotherapy-induced spontaneous celiac trunk dissection in metastatic colon cancer: A case report and literature review

**DOI:** 10.1016/j.ijscr.2025.112051

**Published:** 2025-10-10

**Authors:** Hani Abu-Hijleh, Khaled Sawafta, Tasbeeh Al-Kharraz

**Affiliations:** a Department of Medicine, An Najah National University, Nablus, Palestine; b Department of Radiology, Rafidia Surgical Hospital, Nablus, Palestine

**Keywords:** Celiac artery dissection, Chemotherapy toxicity, Fluorouracil adverse effects, Cetuximab, Vascular injury, Case report, Endothelial dysfunction

## Abstract

**Introduction and importance:**

Spontaneous celiac trunk dissection (SCTD) is a rare (0.08 % of arterial dissections) but potentially life-threatening vascular emergency. It is often underdiagnosed due to its subtle presentation and uncommon nature. While hypertension and connective tissue disorders are known risk factors, the vascular toxicity of chemotherapy is an emerging concern.

**Case presentation:**

We report a case of SCTD in a 59-year-old male with metastatic colon cancer, who developed symptoms during treatment with FOLFIRI and cetuximab. Despite well-controlled hypertension and no significant vascular history, the dissection occurred shortly after chemotherapy administration, Imaging confirmed diagnosis without ischemia.

**Clinical discussion:**

The close temporal relationship between chemotherapy and symptom onset raises the possibility of chemotherapy-induced endothelial injury as a contributing factor. Imaging confirmed the diagnosis, and the patient was successfully managed conservatively without surgical or endovascular intervention.

**Conclusion:**

This case suggests FOLFIRI/cetuximab may trigger SCTD, emphasizing the need for vascular awareness in cancer patients with atypical abdominal pain.

## Highlights

• Spontaneous Celiac Trunk Dissection (SCTD): is a rare vascular event, with chemotherapy-induced endothelial injury emerging as a possible trigger, particularly in patients receiving fluoropyrimidines and EGFR inhibitors.
• Case Presentation: A 59-year-old male with metastatic colon adenocarcinoma developed SCTD after treatment with FOLFIRI and cetuximab, despite controlled hypertension and only mild risk factors.
• Vascular Toxicity of Chemotherapy: Chemotherapy agents like 5-FU, oxaliplatin, and cetuximab are implicated in causing endothelial dysfunction, which increases the risk of vascular events like dissection.
• Management: SCTD can be managed conservatively for stable patients, but endovascular or surgical repair may be required for severe cases. This case highlights the importance of monitoring patients receiving chemotherapy for vascular complications.
• Recommendations: Vigilant monitoring, blood pressure management, and a multidisciplinary approach involving oncologists, vascular surgeons, and radiologists are critical in managing chemotherapy-induced vascular complications.

## 1 Introduction

Spontaneous celiac trunk dissection (SCTD) is a rare but dangerous condition that can lead to significant morbidity and mortality if not promptly diagnosed. It accounts for 0.08 % of all arterial dissections [1].

Traditional risk factors include hypertension, connective tissue disorders, atherosclerosis, and trauma [[2], [3], [4], [5], [6]]. All of these risk factors point to the different things that can lead to SCTD, which shows how important it is to early detect it. and management, particularly in high-risk individuals.

However, emerging evidence has suggested that chemotherapy agents, particularly fluoropyrimidines (e.g., 5-fluorouracil, capecitabine) and epidermal growth factor receptor (EGFR) inhibitors (e.g., cetuximab), can induce endothelial dysfunction, thereby increasing the risk of vascular events like dissection [[7], [8], [9],^17^].

We present a rare case of SCTD in a 59-year-old man receiving FOLFIRI/cetuximab for metastatic colon adenocarcinoma, discussing chemotherapy's role in vascular injury. This report adheres to SCARE 2025 guidelines [10].

## 2 Case presentation

A 59-year-old male with metastatic poorly differentiated colon adenocarcinoma (diagnosed October 2023; T4N1M1 with bladder/liver metastases) presented with abdominal distension, vomiting, and decreased stoma output. He was undergoing his third cycle of FOLFIRI/cetuximab (previously received XELOX plus Cetuximab 3 cycles, last one on 3/2024).

Medical history included:

- Hypertension (controlled with Lisinopril; off medication at presentation).
- 15 pack-year smoking (reduced to occasional use).
- Prior laparotomy with right-sided transverse loop colostomy for malignant bowel obstruction (February 2025).
- Resolved *C. difficile* infection (February 2025, 10 days duration).

## 3 Clinical timeline

• April 15, 2025: Completion of 3rd FOLFIRI/cetuximab cycle
• April 18: Onset of vomiting, abdominal distension, and decreased stoma output
• April 19: Abdominal CT (comparison to pre-chemotherapy CT showed new dissection)
• April 19–22: Conservative management initiated
• May 14: Follow-up CT angiography (complete healing)

On examination, the patient was conscious, afebrile, and hemodynamically stable (BP 130/80 mmHg). His abdomen was soft but distended, with no tenderness or organomegaly. Well-healed laparotomy and inguinal hernia repair scars were noted, along with a functional right-sided loop colostomy. There was no lower limb edema, erythema, or neurological deficit. The patient underwent screening for connective tissue disorders (Marfan, Ehlers-Danlos) via clinical evaluation, which were negative.

Laboratory investigations revealed leukopenia (WBC 3.72 × 10^3^/μL), elevated CRP (145), mild hyponatremia (130 mmol/L), thrombocytopenia (platelets 85 × 10^3^/μL), elevated INR (1.59), Liver enzymes showed mild elevation in ALP (542 U/L) GGT (115 U/L), and pre-renal acute kidney injury (creatinine 1.3 mg/dL, BUN 26 mg/dL).

**Fig. 1:**
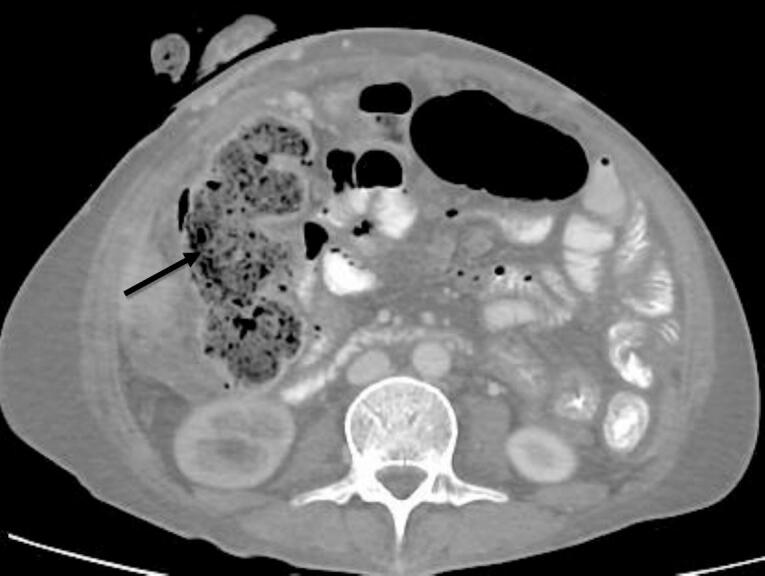
Axial CT. Abdominal CT scan with IV and oral contrast - venous phase, axial view: the colon appears loaded colon (black arrow). Incidentally we found focal aneurysmal dilatation of the celiac trunk measures about 1.3 cm in diameter with evidence of internal dissecting flap.

**Fig. 2:**
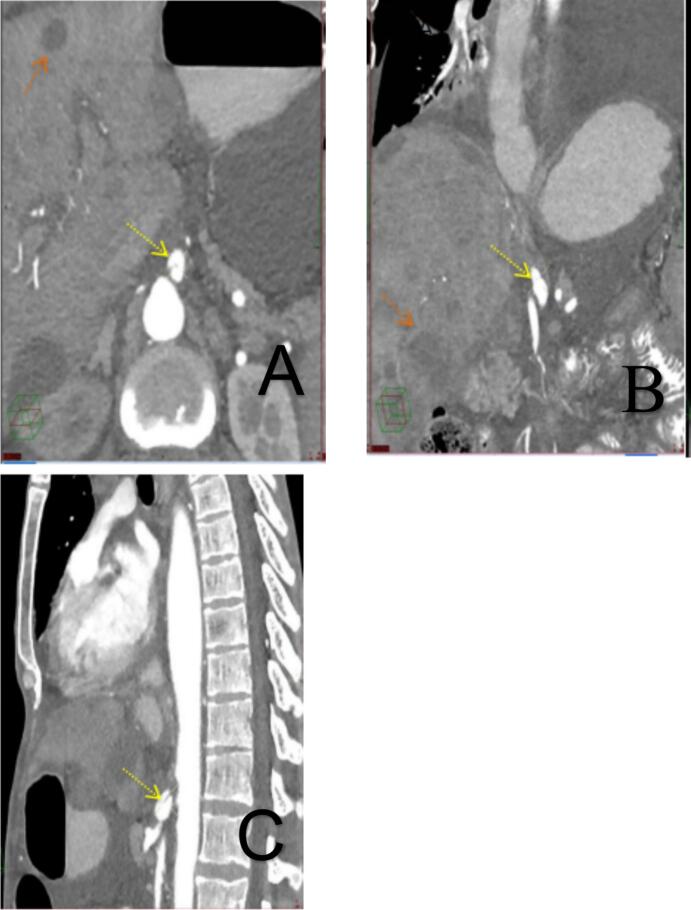
Axial (A), Coronal (B), Sagittal (C) CT showing dissecting flap (yellow arrow). Abdominal CT scan with IV and oral contrast (shown in figure above) - axial, coronal and sagittal views (A, B, C respectively), arterial phase: focal aneurysmal dilatation of the celiac trunk with evidence of internal dissecting flap (yellow arrow) and liver shows multiple metastatic lesions (organ yellow A, B). (For interpretation of the references to color in this figure legend, the reader is referred to the web version of this article.)

CT imaging with IV/oral contrast demonstrated a loaded colon without mechanical obstruction (Fig. 1). Incidentally, a 1.3 cm aneurysmal dilatation of the celiac trunk was identified with an internal dissecting flap (Hounsfield units: −30 to −70) consistent with spontaneous celiac trunk dissection (Fig. 2A-C). No bowel ischemia or rupture was observed.

The vascular surgery team initiated conservative management due to stable hemodynamics, absence of ischemia, thrombocytopenia, and elevated bleeding risk. The protocol included:

1. Intravenous fluid resuscitation (70 cc/h normal saline) with potassium supplementation
2. Lisinopril restart (target SBP <140 mmHg), with strict renal monitoring
3. Enoxaparin 40 mg SC daily for DVT prophylaxis
4. Ondansetron 8 mg IV PRN and paracetamol 1 g IV PRN for symptom control
5. Esomeprazole 40 mg 1*3 IV PRN for gastrointestinal prophylaxis

By April 22, symptoms resolved and renal function normalized (creatinine 0.9 mg/dL). Discharge medications: Lisinopril, low-sodium diet. May 14 CTA confirmed complete healing. A surveillance plan was established with quarterly CT angiography and coordinated oncology/vascular surgery reviews.

## 4 Discussion

Spontaneous Celiac Artery Dissection (SCAD) usually affects men aged 30 to 80 (average age 53). Approximately 31 % of patients had excessive blood pressure, and 23 % were smokers [11,12]. Diabetes and high cholesterol are uncommon, indicating that SCAD is not atherosclerotic [12].

SCTD occurs when an intimal tear in the artery allows blood to enter the vessel wall, creating a false lumen. This leads to compromised blood flow to the abdominal organs supplied by the celiac trunk. The rupture of the dissection can result in life-threatening hemorrhage or organ ischemia.

In this patient, the dissection likely resulted from synergistic effects of chemotherapy (FOLFIRI/cetuximab as they have a known vascular toxicity) [15,19], mild hypertension (contributes to vascular wall fragility overtime even if well-controlled) [20], smoking (As a known risk factor for vascular diseases) [18], and prior abdominal surgery (not directly, but could compromise vascular integrity by altering dynamics of blood flow). Then after Connective tissue disorders were clinically excluded, Notably, the temporal association with chemotherapy and absence of other strong triggers implicate drug-induced endothelial injury as the primary culprit.

To contextualize our case, Table 1 summarizes conservatively managed SCTD cases reported in the last decade, including those associated with chemotherapy.

**Table 1:** Summary of conservatively managed spontaneous celiac trunk dissection (SCTD) cases, including those associated with chemotherapy.

Age/Sex	Trigger/Risk factors	Time to onset	Imaging modality	Conservative management	Outcome	Reference
42 M	None	Abrupt onset	CT abdomen/pelvis	IV heparin, carvedilol (BP <110 mmHg), warfarin 6 mo	Symptom-free; normal CTA at 6 mo	[21]
55 M	None	Acute epigastric pain	CT angiography	Aspirin + beta-blocker	Resolution at 3 wk. CTA; no recurrence	[22]
67 M	Bevacizumab (chemo)	3 d after chemotherapy	Contrast CT scan	BP control (somatostatin, valsartan)	Stable CTA at 3 mo; no ischemia	[23]
59 M	FOLFIRI + cetuximab (chemo)	3 d after chemotherapy	CT angiography	IV fluids, lisinopril (BP control), enoxaparin (prophylaxis), supportive care	Complete healing on 1-mo CTA; resumed modified FOLFOX chemo	Present case (2025)

In the case of this patient, chemotherapy likely played a significant role in the pathogenesis of the dissection. Several chemotherapy agents, particularly 5-fluorouracil (5-FU), oxaliplatin, and cetuximab, have been implicated in causing vascular injury through various mechanisms (Table 2).

**Table 2:** Chemotherapy agents and their vascular toxicity mechanisms.

Agent	Class	Mechanism of vascular toxicity	Reference
5-Fluorouracil (5-FU)	Fluoropyrimidine	5-FU inhibits thymidylate synthase → ↑ Reactive oxygen species → endothelial apoptosis	[7,9,13]
Irinotecan	Topoisomerase inhibitor	Promotes the release of Pro-inflammatory cytokines → matrix metalloproteinases (MMPs) activation → degradation of vascular structural components such as elastic fibers.	[8]
Cetuximab	EGFR inhibitor	Inhibits EGFR-mediated signaling → inhibition leads to increased vascular permeability and delayed endothelial healing after injury.	[7,9]

### 4.1 Why chemotherapy likely contributed to the dissection

While hypertension and smoking are established risk factors for dissection, the patient's well-controlled blood pressure and intermittent smoking history make them an unlikely primary cause in this case. The temporal correlation between chemotherapy administration and the onset of symptoms, coupled with the presence of chemotherapy-induced leukopenia and systemic inflammation (elevated CRP), further supports the role of chemotherapy in the development of the dissection. Literature also supports the association between chemotherapy, particularly 5-FU, and vascular events like dissection [9,15,17].

SCAD treatment may be either conservative or surgically there is no consensus on which approach is ideal based on literature, thus choosing either of them depends mainly on the severity of the dissection and the presence of ischemia:

1. Conservative Management: For stable patients without significant ischemia, conservative treatment, including blood pressure control, pain management, IV fluids and observation, is appropriate. This patient was managed conservatively due to his thrombocytopenia and recent surgery in addition to being mildly symptomatic and stable, which made invasive interventions riskier [7]. Blood pressure control was prioritized using ACE inhibitors due to a compelling vascular indication. Lisinopril was restarted with thrice-daily monitoring of renal function during AKI resolution. The rapid reversal of AKI with IV fluid resuscitation supports the clinical safety of this approach.
2. Endovascular Repair: For symptomatic patients or those with significant branch involvement, endovascular stenting can be an effective minimally invasive option. However, this patient's high INR (1.59) due to coagulopathy made endovascular repair riskier [12,16].
3. Surgical Repair: In cases of rupture or organ ischemia, surgical intervention may be necessary. However, due to the patient's metastatic cancer and the risks of high morbidity, surgery was not indicated in this case [3].

Following resolution of the vascular event, chemotherapy was resumed with a modified FOLFOX regimen excluding anti-EGFR therapy, after a multidisciplinary discussion. Surveillance included quarterly CT angiography during the first year.

## 5 Limitations

This case report is based on a single patient, which limits the ability to generalize the findings to larger populations. Additionally, while CT angiography confirmed the dissection, endothelial biomarkers (e.g., VEGF, endothelin-1) were not measured, which could have provided further insight into the molecular mechanisms at play, Future reports could incorporate measurement of endothelial biomarkers to refine mechanistic understanding and strengthen causal inferences. Finally, the absence of a control group limits our ability to definitively establish causality between chemotherapy and SCTD.

## 6 Clinical recommendations

1. Screen for vascular symptoms during fluoropyrimidine/EGFR inhibitor therapy.
2. Urgent CT angiography for acute abdominal pain in chemotherapy patients.
3. Multidisciplinary management (oncology/vascular surgery/radiology).

## 7 Conclusion

This case suggests FOLFIRI/cetuximab may trigger SCTD. Early imaging and conservative management prevented complications. Vascular monitoring should be prioritized in high-risk regimens.

## Author contribution

Hani Abu-Hijleh: Introduction, Discussion, Conclusion, References.

Khaled Sawafta: Abstract, Case presentation.

Tasbeeh Al-Kharraz: Case presentation.

## Informed consent

Written informed consent was obtained from the patients for their anonymized information to be published in this article.

## Ethical approval

Our institution (An-Najah national university hospital) does not require ethical approval for reporting individual cases or case series.

## Guarantor

Hani Abu-Hijleh.

Khaled Sawafta.

Tasbeeh Al-Kharraz.

## Research registration number

Not applicable.

## Funding

No specific grant from funding agencies was received for this work.

## Conflict of interest statement

The authors state that they have no conflict of interest to be mentioned.
